# A three-terminal ultraviolet photodetector constructed on a barrier-modulated triple-layer architecture

**DOI:** 10.1038/srep26169

**Published:** 2016-05-16

**Authors:** Daqian Ye, Zengxia Mei, Huili Liang, Lishu liu, Yonghui Zhang, Junqiang Li, Yaoping Liu, Changzhi Gu, Xiaolong Du

**Affiliations:** 1 Key Laboratory for Renewable Energy, Beijing National Laboratory for Condensed Matter Physics, Institute of Physics, Chinese Academy of Sciences, Beijing, 100190, People’s Republic of China

## Abstract

We report a novel three-terminal device fabricated on MgZnO/ZnO/MgZnO triple-layer architecture. Because of the combined barrier modulation effect by both gate and drain biases, the device shows an unconventional I–V characteristics compared to a common field effect transistor. The photoresponse behavior of this unique device was also investigated and applied in constructing a new type ultraviolet (UV) photodetector, which may be potentially used as an active element in a UV imaging array. More significantly, the proper gate bias-control offers a new pathway to overcome the common persistent photoconductivity (PPC) effect problem. Additionally, the MgZnO:F as a channel layer was chosen to optimize the photoresponse properties, and the spectrum indicated a gate bias-dependent wavelength-selectable feature for different response peaks, which suggests the possibility to build a unique dual-band UV photodetector with this new architecture.

The classic three-terminal device such as field-effect transistor (FET) is, and always has been, the workhorse of the microelectronics industry. Today, as metal–oxide–semiconductor FET (MOSFET) technology gains a huge commercialized market, coupled with the sharp growth in oxide semiconductors thin film transistor (TFT) for displays, the demand for FETs continues to increase throughout the world-wide microcircuits, and has led to a rapid expansion of microprocessors, memory chips and active matrix *et al.* over the last more than four decades^1^,^2^,^3^.

Despite these advantages of the transistors, the intrinsic advantage of three-terminal device has been proved as one of the best candidates for improving the low power consumption and high-sensitivity ultraviolet photodetectors (UV-PDs) such as ZnO based photodetectors^4^,^5^,^6^. As we all know, different types of UV-PDs based on ZnO have been reported early from 1950s^7^,^8^; however, they mainly utilize two terminal devices, which include photoconductive, Schottky, and p/n-junction types^9^,^10^,^11^,^12^. Moreover, for these structures, there are two critical drawbacks to be overcome before the ZnO UV-PDs constructed on UV imaging or other applications: 1) The persistent photoconductivity (PPC) effect, which was extensively ascribed to the existence of metastable shallow donor state of oxygen vacancy (V_O_) located within the bandgap of ZnO^13^,^14^,^15^,^16^. Especially under the illumination of short-wavelength light, the PPC can cause the semiconductor material to remain conductive for hours/days, even in the absence of light. This increases the response times and limits the frame rates. 2) Low photo gain. A high performance UV-PDs needs high sensitivity. However, due to the high background carrier concentration and low photo gain, most UV-PDs suffer from low discrimination ratio between UV and dark/visible light, which limits the application of UV-PDs^17^,^18^,^19^.

To suppress the PPC effect in oxide UV-PDs and amplify the photo gain, the ability of three terminal photo-TFTs in controlling the position of the Fermi level and carrier densities has been considered. On one hand, Sanghun Jeon *et al.*^5^ and Pavel Reyes *et al.*^20^ found that the capability of the gate bias to tune the current in these three terminal devices can be specifically used in reducing the PPC in GIZO/IZO/GIZO and ZnO, respectively. On the other hand, Bae and T. H. Chang demonstrated a UV sensitive ZnO TFT where the drain current (I_ds_) can be amplified by controlling the gate voltage (V_g_). Therefore, the TFT UV-PD shows a gigantic photoresponsivity and the photo-current is tremendously enhanced. However, visible response was also found when the photon energy exceeds 2.3 eV, which is ascribed to the photo-response from the mid-band gap states^21^.

Differently, in this work, we adopted herein a new three-terminal FET-based UV PD to allow a rapid recovery from the PPC effect, by utilizing its novel biasing control features differentiating from the traditional FETs.

The new three-terminal device consists of a tri-semiconductor MgZnO/ZnO/MgZnO layers synthesized on a p-type Si (111) substrate by radio-frequency plasma assisted molecular beam epitaxy (rf-MBE). Unlike the common bottom-gate FETs, the Ti (20 nm)/Au (50 nm) was deposited on the top MgZnO layer instead of ZnO channel layer to form drain and source electrodes, and indium as the back contact to p-Si for gate electrode [Fig. 1(a)]. Note, the six-fold symmetrical streaky lines recorded by *in-situ* reflection high-energy electron diffraction (RHEED) monitor retained in the whole growth process of all sandwiched layers [Fig. 1(b)], indicating that all triple layers in Mg_x_Zn_1−x_O/ZnO/Mg_x_Zn_1−x_O follow a quasi-homo epitaxy mode with the single-phase wurtzite structure and smooth interface morphology^22^. So the interface defects induced by lattice strains between the sandwich layers are minimal, and the crystalline qualities of these triple thin films can be ensured. The optical band gap of the top, middle and bottom layers is determined as 3.65 eV (339.4 nm), 3.25 eV (382 nm) and 4.02 eV (308.6 nm) by room-temperature photoluminescence spectra (PL), respectively [Fig. 1(c)]. The Mg compositions of the top and bottom Mg_x_Zn_1−x_O layers are hence determined as ~26% and ~40%, respectively. From the peak positions indicated by the arrows in the scanning electron microscopy (SEM) image, the thickness of each layer in the tri-layer architecture is ~150 nm, ~72.8 nm, and ~353 nm for top MgZnO, middle ZnO and bottom MgZnO, respectively [Fig. 1(d)]. In this stacked structure, the bottom Mg_X_Zn_1−X_O layer was employed as dielectric to allow adjustment of the source to drain current which has been reported in our previous work^22^, while the top MgZnO was used to provide Schottky barrier between channel layer and Ti/Au electrode. In addition, it can protect the sandwiched ZnO channel layer from damage during processing.

## Results

**Figure 2:**
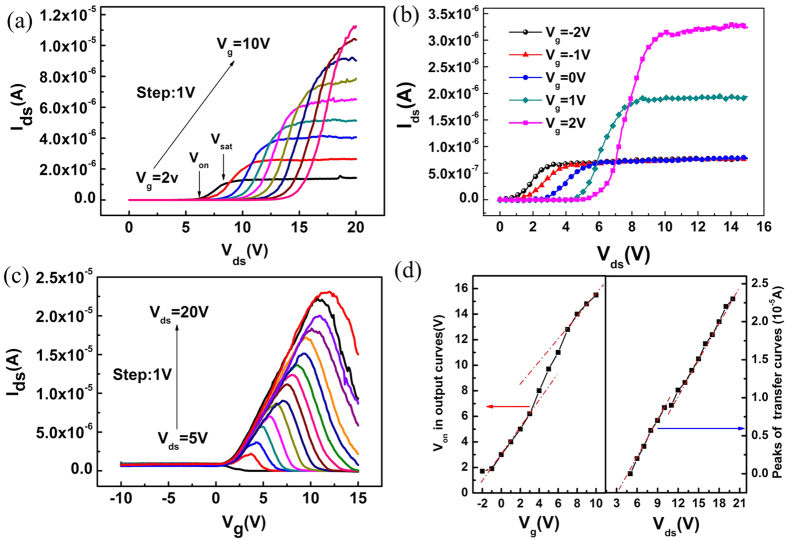
novel I–V characteristics of our three-terminal device (**a**) I_ds_–V_ds_ characteristics of MgZnO/ZnO/MgZnO/Si FET with V_g_ increased from 2 V to 10 V in a forward step of 1 V, (**b**) I_ds_–V_ds_ characteristics conducted over a range of V_ds_ (0 ~ 15 V) and V_g_ (−2 ~ 2 V) (**c**) I_g_–V_g_ characteristics as functions of V_ds_, and (**d**) variation of the peaks in transfer curves and turn on voltages in output curves as a function of V_ds_ and V_g_, respectively.

Compared to conventional ZnO or MgZnO FETs/TFTs with common bottom gate structure^22^,^23^,^24^, the design and fabrication of this three-terminal device owns significantly unconventional I–V characteristics. Firstly, in the output curves (I_ds_–V_ds_) [Fig. 2(a)], the source to drain current (I_ds_) shows an off-state (lower than 1 × 10^−10^A) at the beginning stage of drain voltage (V_ds_). Then, after a turn on voltage (V_on_), I_ds_ goes to linear regions and finally saturates at a high V_sat_. Here, we defined the V_on_ as the V_ds_ that forms a conductive channel between the source and drain electrodes, which lead the I_ds_ change from lower off current to linearly increasing region; and the V_sat_ as the V_ds_ that makes the source to drain current I_ds_ moving from linearly-increasing region to a saturation level. It has been well established that V_on_ is related to the gate voltage, and the saturation current (I_ds_) increases with the gate biases due to the parallel plate capacitor structure formed by the gate electrode, dielectric and semiconductor. To understand the modulation principles of I_ds_–V_ds_ curves, the carrier transport was conducted over a range of V_ds_ (0 ~ 15 V) and V_g_ (−2 ~ 2 V) as shown in Fig. 2(b). When the gate bias raises up from negative bias −2 V to 0 V, V_on_ increased from 1 V to 3 V, but the carrier density finally maximized at the same saturation level. As a comparison, when the gate bias varied from 0 V to positive bias of 2 V, V_on_ increases as usual, but the saturation carrier density dramatically increases to a higher level. Secondly, in the transfer curves (I_ds_–V_g_) as shown in Fig. 2(c), it was observed that the channel layer transformed from the electrons-scattering state to the electron-rich state when V_g_ varied from −10 V to 15 V. In turn, the I_ds_ reached the maximum value after a no-current-flow stage. However, different from the traditional FETs, the source to drain current I_ds_ in Fig. 2(c) dropped to an ‘off current’ instead of keeping increasing at ‘on current’ and tending to be saturated as the gate bias increased. As shown in Fig. 2(d), it is interesting that V_on_ in the output curves (I_ds_–V_ds_) and the peak values of I_ds_ in the transfer curves (I_ds_–V_g_) remained a clearly “s” shape with increasing V_g_ and V_ds_, respectively. These characteristics of three-terminal device are firstly reported. Therefore, the conventional carrier transport theory is not suitable to explain the unique carrier transport behavior of our new device, and a new mechanism is required to interpret such a novel I–V features.

## Discussion

**Figure 3:**
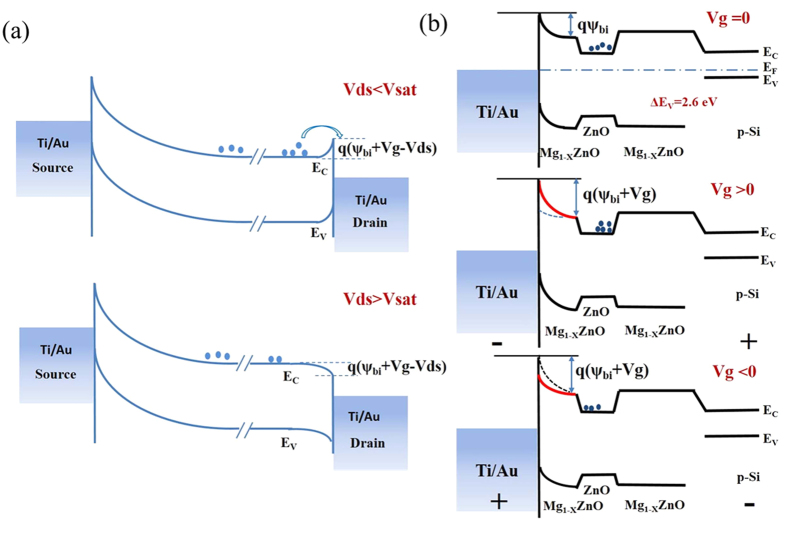
The energy band diagram of MgZnO/ZnO/MgZnO/Si structure: (**a**) illustrated along the source to drain axis for various V_ds_ and V_g_ conditions and (**b**) the energy band diagrams along the drain to gate axis.

To interpret the origin of these novel I–V characteristics, the energy band diagrams in different V_ds_ and V_g_ conditions are illustrated in Fig. 3(a), along the source to drain direction. Firstly, at the gate bias of 0 V, the electrons will drift along the channel driven by a positive V_ds_. As we all know, a Schottky barrier height (SBH) exists at the interface of both source/drain electrodes and the underneath MgZnO layer, mainly induced by the work function difference between MgZnO and Ti^25^,^26^. This SBH can be enlarged by applying a negative V_ds_ or a positive V_g_, respectively. On the other hand, a higher barrier can separate the carriers in ZnO channel layer from the electrodes, which makes the device working in an off state as shown in Fig. 2(a,b). The barrier height Φ_ms_ under the equilibrium condition can be theoretically estimated as 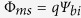, where 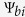 is the build-in potential^27^. Under the conditions of V_ds_ and V_g_ not equal to zero, the barrier height can be written as 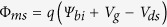. From the equation of 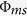, we can see that the barrier height will be reduced as a positive V_ds_ is applied, as shown in the top part of Fig. 3(a). In this case, the electrons can cross the barrier and be collected by the drain electrode to form a very low current flowing between the drain and source (I_ds_) when V_ds_ increases. However, if the V_ds_ is big enough (V_ds_ > V_sat_), the barrier height can be depleted totally, which will result in the I_ds_ going into a saturation region [shown in the bottom part of Fig. 3(a)]. Such processes correspond to the off, linearly and saturation regions in Fig. 2(a,b), respectively. Secondly, the barrier height is also strongly dependent on the gate modulation effect. For the electrons in the channel layer, a lower barrier caused by a negative V_g_ allows them to transport through the barrier under a relatively lower V_on_ [Fig. 2(b)]. Meanwhile, a negative gate voltage can deplete the electrons largely, which makes the saturation currents be kept at a same level at V_g_ values of −1 V and −2 V [Fig. 2(b)]. For a positive gate bias case, however, the higher barrier not only raises V_on_ to a higher level but also gathers a larger amount of electrons in the channel layer, which yields a higher saturation current consequently. Therefore, the channel current in the output curves of the three-terminal device shows a novel gate bias-dependent behavior, which can be delicately modulated by a combination of V_ds_ and V_g_.

Similarly, this gate and V_ds_ controlled barrier-modulation model can also thoroughly interpret the transfer curves shown in Fig. 2(c). From the energy band diagrams along the drain to gate direction illustrated in Fig. 3(b), it can be seen that, if a negative V_g_ is applied, the electrons are repelled from the interface between ZnO and bottom MgZnO layer to establish a depletion layer. Although the barrier height underneath the drain/source is reduced in the negative gate bias case, the channel current continues to be kept at a low level. When V_g_ > 0, the barrier height will be enlarged accordingly, but the electrons are attracted to the interface to form an accumulation layer which can cross over the barrier and give rise to the increased drain current. Further increasing the positive V_g_ may create a barrier high enough to suppress the electrons’ collection at the source electrode, which is identified by a clearly drop in drain current. It should be noted that an optimized condition of a suitable V_g_ can be reached where the barriers for electron are minimized at the source and drain ends concurrently, contributing to an unconventional peak in the transfer curve [Fig. 2(c)]. Because the V_g_ not only controls the SBH obviously but also tunes the electrons attracted in channel layer, which makes the SBH changing as an “s” shaped behavior instead of linearity relation. This combination ability results in the unique I–V characteristics shown in Fig. 2(d).

**Figure 4:**
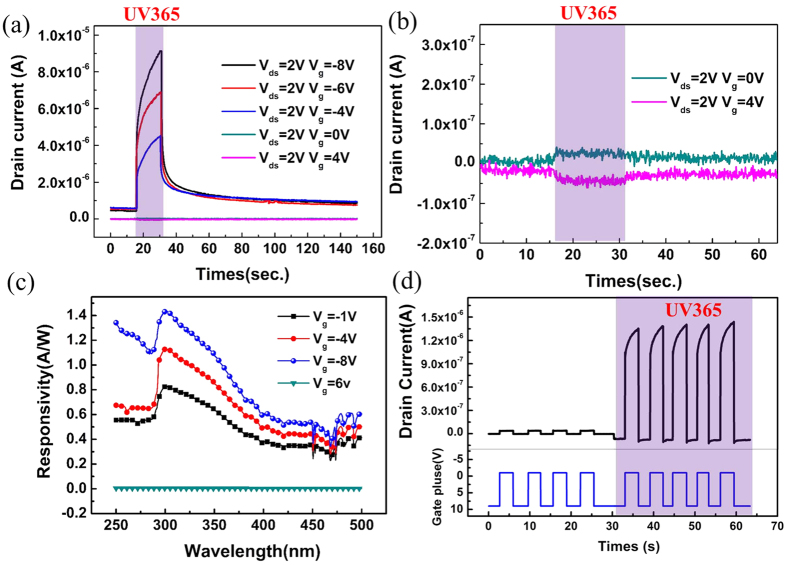
Photoresponse of novel three-terminal device (**a**) Drain–source current as a function of time as the device is subjected to a UV 365 nm light pulse at different gate biases V_g_ varied in a range of 4 V to −8 V, (**b**) amplification of drain-source current under gate voltages V_g_ = 0 V and 4 V, (**c**) The room-temperature spectral responsivity of the MgZnO/ZnO/MgZnO/Si PD under different bias voltages, and (**d**) novel UV PD. The V_ds_ was kept at 2 V bias to monitor the electrical and optical characteristics, a continuous V_g_ plus was applied varied between −1 V and 9 V. After the illumination of UV 365 nm light, the large gain of photocurrent were nicely observed.

**Figure 5:**
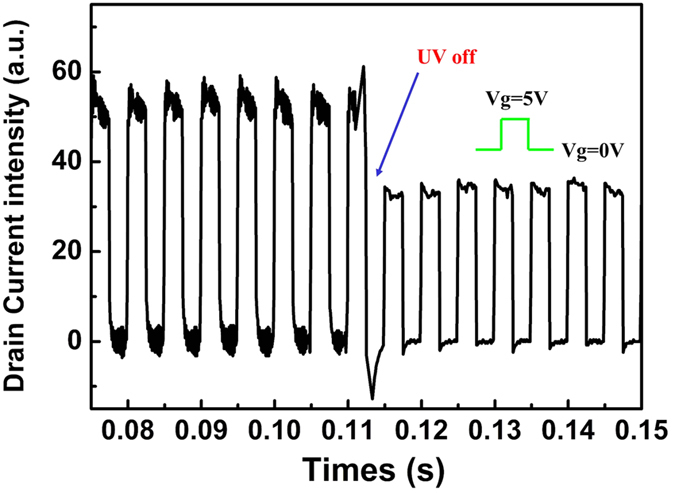
suppression of the PPC when the PD is pulsed with positive gate bias.

In order to thoroughly explore the unique merits of this drain electrode barrier engineered three-terminal device, its photoresponse properties were investigated when the device was subjected to a light pulse. As shown in Fig. 4(a), a pulsed 365 nm illumination was applied when the photo-device was biased on a V_ds_ of 2 V and V_g_ varied from −8 V to 4 V, respectively. Here, the positive V_ds_ is to read out the data and V_g_ is biased under different biases to check the photocurrent gain feature. Obviously, it is the negative gate bias that greatly enlarges the photo gain. This relation will be discussed and shown later. At the same time, there is a slow UV response in UV-PDs, which may be attributed to the oxygen adsorption and desorption processes^28^,^29^,^30^. In dark, oxygen molecules adsorb on the ZnO surface at the surface states by capturing free electrons from the n-type ZnO, thereby creating a depletion layer with low conductivity near the surface. Upon UV illumination at photon energies above ZnO band gap, electron hole pairs are generated. Photon-generated holes migrate to the surface and discharge the adsorbed oxygen ions to photon-desorbed oxygen from the surfaces. The unpaired electrons accumulate gradually with time until desorption and re-adsorption of O_2_ reach an equilibrium state, resulting in a gradual current rise until saturation during UV illumination. As demonstrated in Fig. 4(b), due to the barrier height existing underneath the drain side, no photocurrent can be detected in drain electrode although the device is exposed to a UV light of 365 nm. A positive gate voltage will elevate the height value and induce a suppressed photocurrent even lower than dark current. The photoresponse spectrum was measured with a fixed V_ds_ (7 V) and the light wavelength ranges from 250 to 500 nm [Fig. 4(c)]. The gate voltage was set as −1 V, −4 V, −6 V, −8 V and 6 V, respectively. It was found that when a negative gate voltage was applied, a photocurrent peak can be clearly recognized at 295 nm, and the responsivity value significantly increased to 1.45 A/W at V_g_ of −8 V, indicating the existence of an internal gain in the device. It can be clearly seen that the gap of tested photocurrent between V_g_ at −4 V and −8 V is larger at 250 nm. We think that this mechanism is similar to MgZnO MSM UV-PDs^17^,^31^,^32^. In the UV light region, the remnant photo-induced carriers can lower the built-in potential and thus the Schottky barrier, which allows the electrodes to emit more electrons. On the other hand, the SBH underneath the drain/source is also reduced in the negative gate bias case. These two reasons dominated the photo-current inverting to a higher level when a lager negative gate bias was applied. In contrast, when a positive gate voltage was applied, the responsivity was almost independent of the bias, implying that the gain was much smaller than the negative case. The approach to the control of photocurrent gain through the proper gate biasing provides the device a big potential to be used as an active UV PD element in UV imaging arrays. Such a configuration possesses the intrinsic advantage as a three-terminal PD, where UV illumination can serve as the “write” function, the positive gate bias as the “clear” function, and the negative gate bias as the “store” function. For this purpose, as demonstrated in Fig. 4(d), when the V_ds_ was kept at 2 V to monitor the electrical and optical characteristics, a continuous V_g_ pulse was applied varying between −1 V and 9 V. Under the dark conditions, the carrier-confinement ability of the drain/source SBH tuned by the negative/positive gate voltage dominated the drain to source current (I_ds_) as shown in Fig. 3(a), so the drain current alternatively stayed at a high level (~4 × 10^−8^A) with V_g_ of −1 V and low level (~7 × 10^−9^A) with V_g_ of 9 V, respectively. However, under UV 365 nm illumination, it can be seen that in channel layer there is a large gain and enhanced the photocurrent to 1.3 × 10^−6^ A be detected. At V_g_ of −1 V, the photocurrent is two order of magnitudes lager than dark current, which is preferred to act as an ideal UV PD. To explain this photo-generated response processes mechanism, a reduced-Schottky barrier height (SBH) model can be adopted^31^,^32^,^33^. Similar to MgZnO MSM UV-PDs, in the UV light region, the remnant photo-induced carriers can lower the built-in potential and thus the Schottky barrier, which allows the electrodes to emit more electrons. Moreover, the positive gate biasing can deplete the carrier in channel layer completely and timely, which provides us another pathway to overcome the PPC problem in ZnO and GaN related devices^13^,^34^. As shown in Fig. 5, a pulsed gate voltage was exerted changing from −1 V to 9 V with a frequency of 100 Hz. After the UV light was shut off, the photocurrent rapidly decayed in less than 5 ms due to the remarkable suppression effect of positive gate biasing control. In GIZO/IZO/GIZO structure, the all-oxide photo sensor array prototype based on the bias-assisted PPC recovery scheme shows a recovery time about 0.25 ms^5^; In ZnO UV PD based on a thin film transistor with a back gate configuration, the recovery time is ~5 ms^20^. Our three terminal device based on MgZnO/ZnO/MgZnO triple-layer architecture shows a recovery time less than 5 ms. It can be seen that our FET-based UV-PD is better than ZnO but worse than GIZO/IZO/GIZO, which may be ascribed to the shorter band gap and lower oxygen vacancy (V_O_) density in GIZO/IZO (2.9 eV)/GIZO.

**Figure 6:**
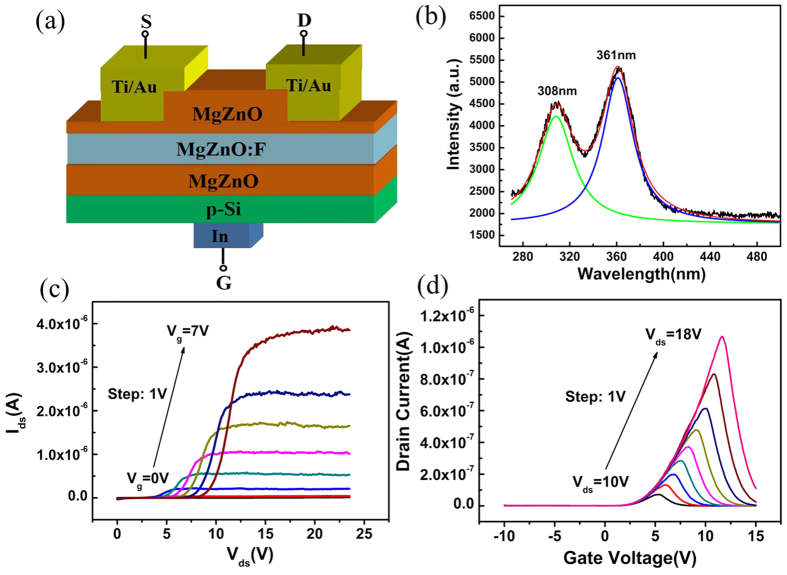
The structure and electrical characteristics of a novel three-terminal device: (**a**,**b**), schematic diagram and PL spectrum of novel three-terminal device using MgZnO:F as channel layer; (**c**) and (**d**), output and transfer curves of novel three-terminal device using MgZnO:F as channel layer.

Another parameter to evaluate a UV PD is the cutoff edge in photoresponse spectrum^12^. However, as shown in Fig. 4(c), the cutoff is not that sharp because of the different band gap energies of top-MgZnO, ZnO and bottom-MgZnO layers. In order to improve this flaw, ZnO channel layer was replaced by MgZnO doped with F ions [Fig. 6(a)]^35^,^36^. On one hand, the triple layers have similar bandgap as shown in photoluminescence (PL) spectrum [Fig. 6(b)]. The two colored curves are the fitting results of the PL spectra (black line). It can be seen clearly that two fitting curves are located at 308 nm (bottom-MgZnO and MgZnO:F ) and 361 nm (top-MgZnO), respectively, corresponding to the band edge emissions from three different epitaxial layers; on the other hand, the carrier density of MgZnO:F layer is about ~2 × 10^17^/cm^3^, which will not influence the I–V characteristics substantially. The I–V curves of three-terminal device based on MgZnO/MgZnO:F/MgZnO layers deposited on Si are shown in Fig. 6(c,d). The similar transfer and output curves imply a same carrier transport mechanisms in this structure as that of MgZnO/ZnO/MgZnO on Si.

**Figure 7:**
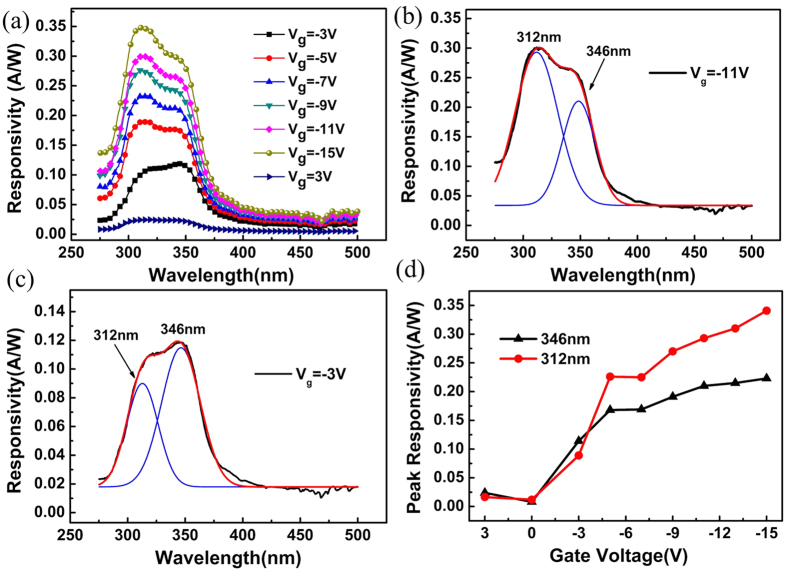
Photoresponse of novel three-terminal device (**a**) the room-temperature spectral responsivity of the MgZnO/MgZnO:F/MgZnO/Si PD under different bias voltages, (**b**,**c**) the Gauss fitting of photoresponse curves, and (**d**) the peak responsivity of two wavelengths in the new type PD under different biases.

The interesting photoresponse features of this three-terminal device were shown in Fig. 7(a). Firstly, under the negative gate bias, there are two photoresponse peaks at 312 and 346 nm. Furthermore, a sharp cutoff wavelength at 360 nm can be clearly recognized, which demonstrated the success of our device design in improving the cutoff edge by using MgZnO:F channel layer. In the case of the positive gate bias, a weaker photoresponse was observed due to the elevated barrier under the drain electrode side as we have demonstrated in Fig. 4(c). Secondly, it is worth highlighting that the negative gate voltages show a wavelength-selectable feature in the response peaks. For example, from the Gaussian fitting of the photoresponse curves [Fig. 7(b,c)], the response peaks at 312 nm and 346 nm show different intensity ratio under a −11 V and −3 V gate bias, respectively. As shown in Fig. 7(d), the relation of peak responsivity vs gate voltage is carefully examined in a gate bias range of 3 V~ −15 V. Obviously, the −3 V gate bias is a dividing point. The responsivity of the 312 nm peak increases rapidly than the peak of 346 nm when the gate bias is lower than −3 V, indicating that the negative gate bias enhances the internal gain of this wavelength. The accumulated electrons in MgZnO:F layer were believed to contribute to this increasing responsivity with negative gate bias increased.

In summary, a new three-terminal device based on MgZnO/ZnO/MgZnO triple architecture was fabricated on Si substrate. The devices show novel I–V curves different from the conventional FETs, which is ascribed to the gate and V_ds_ controlled barrier modulation effect. The unique characteristics of such a device configuration were furthermore applied in a new UV PD, where UV illumination will serve as the “write” function, the positive gate bias as the “clear” function, and the negative bias as the “store” function. Beyond that, we found the photocurrent can be tuned in a wide range by the gate voltage, and the quite common PPC effect in ZnO-based PDs can be simultaneously suppressed effectively. In addition, we used MgZnO:F layer instead of ZnO to act as the channel layer, successfully achieving a much sharper cutoff edge in the photoresponse spectra. A gate voltage-dependent wavelength-selectable behavior was revealed for different response peaks, which suggests the possibility to build a unique dual-band UV PD with this new architecture.

## Methods

The MgZnO/ZnO/MgZnO sandwich structure is grown by radio-frequency plasma assisted MBE (rf-MBE) on a p-Si (111) with a resistivity of 0.1 ~ 1 Ω cm, which serves as the common gate and as the substrate. To get a high-quality MgZnO layer with high Mg content, a 3 nm wurtzite BeO interfacial layer was firstly formed on Si by oxidation of Be to protect the clean Si surface and provide a good template for wurtzite MgZnO epitaxy. A 40 nm MgZnO buffer (B-MgZnO) layer with low Mg content was deposited subsequently in order to relax the big lattice strain between the substrate and the following high-Mg-content MgZnO epilayer (300 nm). The ZnO channel layer (~70 nm) with an electron concentration of 2.04 × 10^17^ cm^−3^ was then grown on MgZnO. After that a 150 nm thick MgZnO layer was deposited on top.

The three-terminal devices were fabricated by the standard photolithograph and lift off technique. Before the Ti (20 nm)/Au (50 nm) was deposited to form drain and source electrodes by thermal evaporation, about 100 nm top-MgZnO layer in the electrodes positions was etched by top-down dry etching method using Cl_2_ and BCl_3_ as specific gases. The active layer is fixed with a width/length ratio (W/L) of 150 μm/10 μm.

All the electrical tests are conducted using an Agilent B1500A semiconductor parameter analyzer. The photoresponsivity and photocurrent were measured using a 75 W xenon lamp, followed by a 0.05 nm high-resolution monochromator (SpectraPro-500i, Acton Research Corporation) as the illumination source.

For the photoluminescence measurements, the samples were excited using a Ti:Sapphire laser. The output wavelength is 240 nm and the output energy can reach GW magnitude after focusing to a spot of approximately 2 mm to satisfy the high absorb coefficient of ZnO and MgZnO layers. The spectra were measured at room temperature in a back-scattering geometry.

## Additional Information

**How to cite this article**: Ye, D. *et al.* A three-terminal ultraviolet photodetector constructed on a barrier-modulated triple-layer architecture. *Sci. Rep.* **6**, 26169; doi: 10.1038/srep26169 (2016).

## Figures and Tables

**Figure 1:**
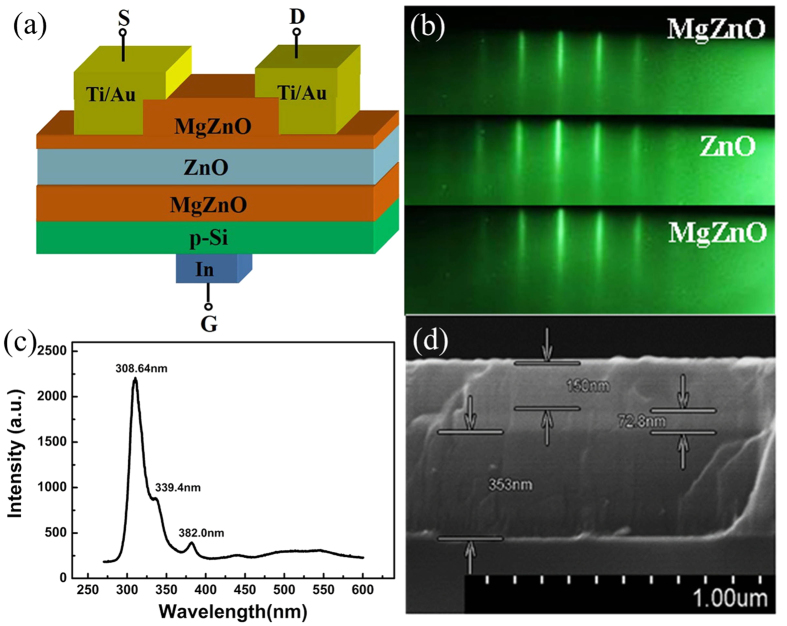
The structure of a novel three-terminal device (**a**) schematic diagram of the device based on MgZnO/ZnO/MgZnO/Si structure, (**b**) RHEED patterns of top MgZnO, ZnO and bottom MgZnO layers as recorded in sample synthesis, (**c**) photoluminescence spectra (PL) of triple layers, and (**d**) the cross-sectional view of scanning electron microscope (SEM) image.
